# Ventilation Design of an Extra-Long Single-Bore Double-Track Railway Tunnel with High Traffic Density

**DOI:** 10.3390/s25134009

**Published:** 2025-06-27

**Authors:** Xiaohan Chen, Sanxiang Sun, Jianyun Wu, Tianyang Ling, Lei Li, Xianwei Shi, Haifu Yang

**Affiliations:** 1 School of Environmental and Municipal Engineering, Lanzhou Jiaotong University, Lanzhou 730070, China; chenxiaohan@stu.lzjtu.edu.cn (X.C.); wujianyun@mail.lzjtu.cn (J.W.); 2 China Railway First Survey and Design Institute Group Co., Ltd., Xi’an 710043, China; gz_lilei.yy@crcc.cn; 3 Guangzhou Municipal Housing Development and Transportation Bureau in Baiyun District, Guangzhou 510405, China; lingty@hnu.edu.cn; 4 China Railway 18th Bureau Group Co., Ltd., Tianjin 300222, China; yanghaifu.18g@crcc.cn

**Keywords:** railway tunnel, diesel locomotives, single-bore double-track tunnel, ventilation design, scale model test

## Abstract

Harmful gases produced by diesel locomotives tend to accumulate within tunnels, posing risks such as dizziness, vomiting, coma, and even death to the working staff, particularly in long tunnels with high traffic density. As the number of such structures increases, ventilation in extra-long tunnels represents a critical challenge within the engineering area. In this study, the ventilation of an extra-long single-bore double-track tunnel operating with diesel locomotives is investigated. Through scale model tests and based on the inspection sensor data, the natural diffusion patterns of harmful gases under various operating conditions were elucidated. Based on the local resistance coefficient optimization theory and numerical simulations, the ventilation shafts of the tunnel were optimally designed, and an overall ventilation scheme was developed. The ventilation effect of the tunnel was verified through improved scale model tests. The results show that harmful gases primarily diffuse towards the higher elevation tunnel entrance, with only gases near the lower entrance escaping from it. Under the same operating conditions, NO_2_ diffuses more slowly than CO, making it harder to discharge. Applying the local resistance coefficient optimization theory, the inclined and vertical shafts of the tunnel can be effectively optimized. The optimized ventilation shafts, coupled with jet fans, can reduce harmful gas concentrations below safety limits within one minute. The methodologies and findings presented here can offer valuable guidance for the ventilation design of similar infrastructures.

## 1. Introduction

Tunnel ventilation plays a crucial role in ensuring the health and safety of workers engaged in tunnel engineering [1]. When the concentration of harmful gas in tunnels exceeds a certain limit, it may cause headache, dizziness, vomiting, coma, and even death of the workers [2,3,4,5]. With the advancement of tunneling technology, the number of extra-long tunnels has gradually increased in recent years [6,7,8,9,10], and how to effectively design the ventilation of extra-long tunnels has become a hot research topic in the engineering field.

Many scholars [11,12,13,14] have studied tunnel ventilation over the past few decades. Theoretical analysis [15,16], numerical simulation [17,18,19,20], and experimental investigation [6,9,21] are common approaches to studying such issues. Generally, obtaining the distribution pattern of harmful gas in tunnels is an essential prerequisite for the rational design of tunnel ventilation. For a tunnel in the Guizhou province of China, Zhang et al. [22] investigated the transport patterns of the airflow, dust, and gas during tunneling by using CFD numerical simulation and verified the simulation results by establishing a physical experimental platform. In that study [22], harmful gas accumulation was observed, and the harmful gas was found to be mainly concentrated in the back-wind side of the tunnel, roughly at 40–60 m from the head of the tunnel. Some scholars have tried to use theoretical models to describe the diffusion pattern of harmful gas in tunnels. For example, Feng et al. [23] proposed a one-dimensional non-constant flow model to calculate the airflow velocity and concentration distribution of harmful gas in tunnels. Then, based on the numerical simulation on the Xinlongmen Tunnel, the hazards of harmful gases produced by diesel locomotives and their composition were described, the amount of discharges of diesel locomotives according to the traction power was also calculated, and the factors affecting the concentration distribution of harmful gas were also investigated. Finally, through a comprehensive comparison of the meeting position of diesel locomotives in the tunnel, the most favorable operating conditions were determined, and the best ventilation scheme was proposed. On that basis, the working time of the jet fan and the direction of the fan’s air supply were rationally determined. Chen et al. [24] proposed a model to describe the harmful gas concentration distribution in railway tunnels according to the atmospheric diffusion equation and used the finite volume method to solve the model. It was found that the emission strength of the harmful gas generated from diesel locomotives and the airflow velocity in the tunnel are the key factors affecting the distribution of harmful gas in railway tunnels. Then, based on the simulation and analysis of the Yangbajing No.1 Tunnel of the Qinghai–Tibet Railway, it was found that the maximum value of NO_x_ concentration in the tunnel often appears around the location of the locomotive head. For these models, airflow velocity is the basis for the calculation of gas concentration and distribution in the tunnel. In the traditional constant airflow theory, after the fan starts, the transition time for the natural airflow velocity in a tunnel to reach the wind velocity of the fan is ignored. The assumption is not reasonable, since the transition process actually existed, which will make the calculated airflow velocity higher than the actual airflow velocity, resulting in a false increase in the kinetic energy of the airflow. As a result, the ventilation time and the amount of the ventilation gas estimated will be smaller than the actual results.

Since harmful gases are prone to being spatially concentrated, adding reasonable ventilation shafts is especially necessary. Commonly, ventilation shafts are opened in the lateral or vertical direction. To study the influence of air intake of inclined shafts on the comfort and aerodynamic safety of train operation, Huang et al. [25] conducted a numerical simulation on the ventilation system of an existing tunnel. Several working conditions (i.e., inclined shafts closed, inclined shafts open, no air intake, and air intake) were considered, and the transient pressure and the aerodynamic force (i.e., lift coefficient, lateral force coefficient, and overturning moment coefficient) were investigated. Zhang et al. [26] conducted a series of fire experiments in a 1:10 scale tunnel model with side-opening shafts and analyzed the favorable effects of a mechanical exhaust system with side-ventilating shafts on the tunnel’s pollutant concentration and longitudinal temperature attenuation. Zhu et al. [27] conducted a series of experiments using a typical small-size shaft test platform in the State Key Laboratory of Fire Science; the smoke movement patterns under different shaft operating conditions were investigated, and the extent of the influence of different shaft lateral opening positions on the ventilation effect was also revealed. Li et al. [28] used numerical calculation methods to simulate the transient pressure of a high-speed train passing through a tunnel with a vertical shaft; the transient pressure history and change amplitude of the car body pressure were measured. It was found that the vertical shaft can effectively alleviate the amplitude of the transient pressure of the car body when the high-speed train passes through the tunnel. Based on non-stationary *N-S* equations and compressible three-dimensional numerical simulation, Yang et al. [29] studied the train body pressure change when the train passes through a tunnel at high speed with or without a vertical shaft. It was found that there is an optimal location of the vertical shafts when the number of vertical shafts and the cross-sectional area of vertical shafts in a tunnel are determined. Under this layout, the mitigating effect of the tunnel on the transient pressure of the train body is the best. Ji et al. [30] used Response Surface Methodology to optimize ventilation efficiency in tunnel-type underground spaces, finding that negative pressure ventilation with multiple shafts performs best. Similar methodology was also applied by Li et al. [31] in the optimization of air distribution of underground building ventilation systems. Wang et al. [32] carried out unsteady state numerical calculations of the tunnel shafts at different locations by means of moving grid and slip-interface techniques and found that train operation mode should be taken into account. The shafts should be set up within the interval section of the train traveling at a constant speed and close to the entrance of the tunnel.

With the advancement of ventilation technology and the increased ventilation requirements, mechanical ventilation has become the mainstream solution in the design of modern tunnel ventilation systems. Zarnaghsh et al. [33] conducted a large number of numerical simulations for a long tunnel equipped with jet fans. It was found that the direction of jet fan airflow and the direction of train travel will jointly affect the airflow direction in the tunnel, which will, in turn, affect the ventilation effect. A similar pattern was found by Król et al. [21]; they conducted full-size field measurements of airflow velocity in a highway tunnel. Egilsrud et al. [34] combined programming to simulate tunnel ventilation and other parameters to obtain the ventilation effect of harmful gas at different intensities with different ventilation systems. Lowndes et al. [35] controlled the fan turn-on by using binary coding and hybrid coding to study the optimal turn-on combinations in the ventilation process and the minimum power. Hu et al. [36], based on a study of ventilation in the case of a mine fire, found that mechanical ventilation equipment, although helpful in ventilation, is not better with more power under the same conditions. In some cases, the higher fan capacity may cause a fire accident in the mine to be more serious [14]; thus, the setting of mechanical ventilation should also be considered during the development of emergency plans.

Based on the above studies, the following can be found: (1) Although theoretical research and numerical simulation techniques have made great progress, for the ventilation design of each specific tunnel, physical experimental tests are still needed. (2) The actual ventilation effect is related to many factors, such as traffic conditions, the surrounding climate environment, ventilation equipment, and the tunnel structural design (shafts). Side-opening shafts are conducive for the tunnel air intake and are also beneficial to the driving comfort and pneumatic safety of the trains, while the vertical shafts have an important role in easing the wind pressure and discharging the harmful gas. (3) For the optimization of ventilation efficiency, some methods, like Response Surface Methodology, were adopted [30,31]. However, these studies are more focused on determining the position of the shafts; little attention has been paid to the optimization of the ventilation shaft itself, and there is usually an optimal strategy for the deployment of the shafts. (4) Mechanical ventilation equipment is the key to improving the ventilation effect, but it is still necessary to consider energy saving and emergency management.

In this paper, the ventilation of an extra-long single-bore double-track railway tunnel is investigated by combining scale model tests with numerical simulation. Firstly, a scale tunnel model was designed and constructed (in Section 3) according to the real sizes of the tunnel introduced in Section 2, to test the diffusion law of the harmful gas in the tunnel under different train operating conditions. Then, the ventilation structures (ventilation shafts and inclined shafts) of the tunnel were optimally designed based on the resistance coefficient optimization theory and numerical simulation (in Section 4), and the ventilation system was determined by combining the mechanical ventilation equipment. Finally, the effect of the designed ventilation scheme was verified (in Section 5) through the modified scale model tests.

## 2. Engineering Background

### 2.1. Tunnel Structures

The newly built railway is located in Guinea, Africa. It is an important railway line for the external transportation of Guinean iron ore, which has important strategic significance for stimulating the import and export trade of Guinean goods and promoting the economic development of Africa.

**Figure 1:**
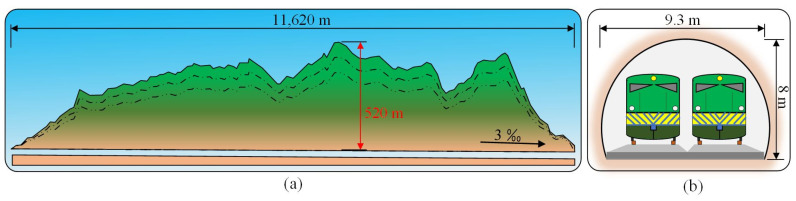
Diagram of the tunnel analyzed: (**a**) tunnel profile; (**b**) cross-section of the tunnel.

The tunnel analyzed in this study, as shown in Figure 1, is one of the critical control projects of the newly built railway. The tunnel has a total length of 11,620 m, and it used to be the longest single-hole, double-track railway tunnel in the world. The maximum overburden depth of the tunnel is approximately 520 m. Both the tunnel entrance and exit are situated in pristine forest areas with well-developed vegetation and poor accessibility. The elevation of the rail surface at the entrance is 96.59 m, while the elevation of the rail surface at the exit is 131.45 m. The longitudinal slope of the tunnel is designed at +3.0‰. The cross-sectional width is designed to be 9.3 m, and the height is 8.0 m, allowing for side-by-side bidirectional train traffic, as shown in Figure 1b.

### 2.2. Traffic Operation Condition

Power infrastructure is underdeveloped in the construction area. Given the advantages of diesel-hauled traction, such as lower capital investment, reduced locomotive procurement costs, independence from external power sources, and minimal requirements for supporting infrastructure, freight trains on this railway line are all hauled by a 6000-horsepower AC diesel locomotive. The harmful gases emitted by this kind of train primarily include Carbon Monoxide (CO), NO_x_, PM, HC, and smoke and dust. According to the “Exhaust Emission Tests for Diesel Engines of Motive Power Units (TB/T 2783-2017)” [37], the specific pollutant emission standards can be calculated according to Table 1. For dual-track operation, the emission levels should be considered at twice the limit values specified in the standards [37].

To meet the enormous freight demand, the average speed of the trains reaches 55 km/h, and the time interval between each train is only 12 min. Considering the most unfavorable operating conditions for emission accumulation, it is possible for up to three diesel-hauled locomotives (six in total, considering the dual-track layout) to be simultaneously operating within the tunnel, continuously emitting harmful gases. This presents significant challenges for the tunnel’s operational management and the ventilation system design.

### 2.3. Natural Wind Condition

**Figure 2:**
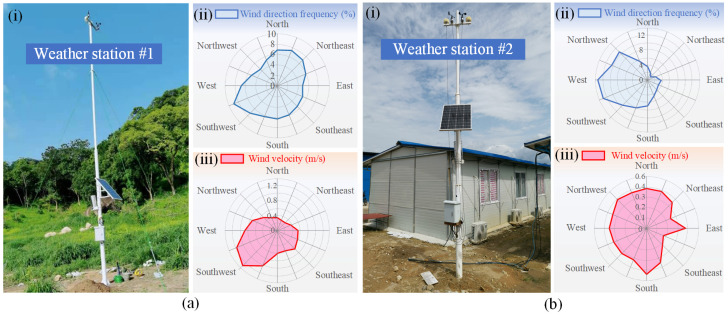
Wind direction and wind velocity measured at the entrance and exit of the tunnel: (**a**) tunnel entrance monitoring—(**i**) photograph of the monitoring site, (**ii**) wind direction plot, (**iii**) wind velocity plot; (**b**) tunnel exit monitoring—(**i**) photograph of the monitoring site, (**ii**) wind direction plot, (**iii**) wind velocity plot.

The natural wind around the entrances of the tunnel has a significant impact on the design of tunnel ventilation. To develop an economically feasible ventilation strategy, two weather stations were set up at the entrance and exit of the tunnel, respectively, to continuously monitor wind velocity, temperature, and humidity in the surrounding area, as shown in Figure 2. Table 2 presents the statistical analysis of monitoring data from September 2021 to April 2022. Based on the results presented in Figure 2 and Table 2, it can be found that while the wind direction in the location area of the tunnel shows slight variations between the dry and rainy seasons, the prevailing wind is generally from the southwest. The maximum wind velocity at the tunnel entrance does not exceed 1.2 m/s, and the overall wind velocity is low, making it insufficient to meet the auxiliary ventilation requirements of the tunnel. Therefore, it is necessary to develop a rational ventilation system to enhance the ventilation performance of the tunnel.

The maximum wind velocity during the observation period was 16.1 m/s (12 September 2021). The highest temperature during the observation period was 41.9 °C (16 April 2022); the lowest temperature was 14.2 °C (29 December 2021), with a relative temperature difference of 27.70 °C.

### 2.4. Ventilation Control Standard

As it is a typical workplace, the ventilation control standards of the tunnel need to be determined according to relevant workplace regulations. Based on the “Occupational Exposure Limits for Hazardous Agents in Workplaces (JTG D70-2004)” [38], the permissible concentration of diesel locomotive exhaust gases in the air within the tunnel in Guinea can be determined as follows:

- For NO_x_ (expressed as NO_2_ in this study), the maximum permissible exposure limit is 10 mg/m^3^, and the average concentration of exhaust gases during any 30 min exposure may not exceed 30 mg/m^3^.
- For CO, the maximum permissible exposure limit is 40 mg/m^3^, and the average concentration of exhaust gases during any 30 min exposure may not exceed 100 mg/m^3^.

## 3. Harmful Gas Distribution Tests

### 3.1. Scale Model Construction

**Figure 3:**
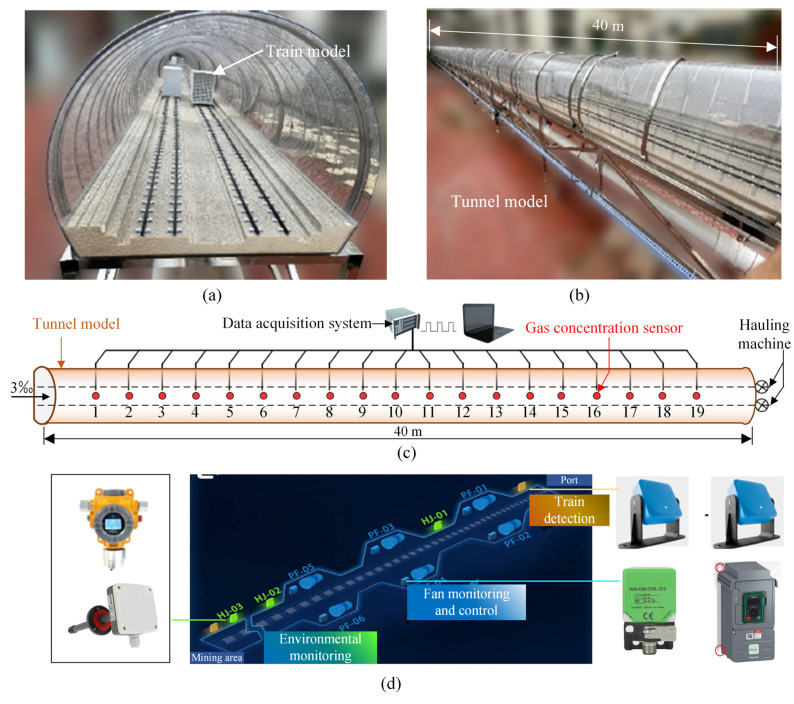
Tunnel model design: (**a**) cross-section view; (**b**) side view; (**c**) layout of the gas concentration sensors; (**d**) inspection system.

To investigate the diffusion pattern of the harmful gas generated from the diesel locomotives within the tunnel, in this chapter, a scale tunnel model was designed and constructed according to the characteristics of the actual tunnel structure based on the similarity principles, as shown in Figure 3. The similarity of total resistance is an important part of dynamic similarity. Dynamic similarity implies that the model and the prototype have similar motion states under the action of forces. In this study, the scaled-model experiment was initiated to investigate the problem of harmful gas diffusion in tunnels. By controlling the similarity of total resistance, the flow characteristics of the fluid in the model and the prototype can be ensured to be similar. Consequently, the model test can accurately simulate the fluid-dynamic phenomena in the prototype.

#### 3.1.1. Tunnel Model Design

Based on the principles of total resistance similarity and modified model theory, the basic length of the tunnel is considered according to a single set of niche jets. The lateral scale of the model is 1:16, and the longitudinal scale of the model is 1:300, with a longitudinal length of 40 m and a tunnel slope of 3‰, as illustrated in Figure 3a,b. The longitudinal frictional resistance inside the tunnel is simulated by setting up ventilation grilles. The frictional coefficient of the model is 0.031, and the friction resistance modification rate of the model is 1.94. The local resistance coefficients at the tunnel entrances and exits are 0.6 and 1.0, respectively. The tunnel is equipped with 12 smoke exhaust inlets: 6 for upward flow and 6 for downward flow. Diesel engine exhaust is introduced into the fan, which then transports it through pipes to the exhaust outlets, simulating the cumulative harmful gas emissions from the diesel locomotive at that position. By controlling the exhaust inlet valves, different operating conditions for the diesel locomotives are simulated. Using a static model, the passage of a diesel locomotive through the tunnel is simulated, with CO and NO_2_ as tracer gases. Continuous monitoring is conducted at various cross-sections of the tunnel to analyze the concentration distribution and diffusion changes of the harmful gas within the tunnel.

#### 3.1.2. Layout of the Gas Concentration Sensors

A set of three sensors (wind velocity, CO, and NO_2_) is arranged at each cross-section of the tunnel. The first set is positioned 1 m from the high tunnel entrance (equivalent to approximately 30 m), and subsequent sets are spaced 1.8 m apart (equivalent to approximately 54 m), with a total of 19 sets. The CO and NO_2_ sensors are installed at the top of the tunnel, while the wind speed sensors are placed at the average wind velocity position of the cross-section. The gas concentration measurement sensors were point-type gas detectors produced by Beijing Kunlun Zhongda Sensor Technology Co., Ltd. (Beijing, China). The flow velocity sensor is of model SM3788B, with a power supply of DC12-24V, a flow velocity range of 0~30 m/s, and an output signal of RS485. The sensor layout and data acquisition system schematic are shown in Figure 3c,d.

The data collection of each sensor and the control of all air dampers and fans in the physical model were centrally controlled by the control room of the test platform. The 19 groups of sensors were divided into two queues, which were controlled by the master-station program and the slave-station program, respectively. Each sensor was connected to the environmental monitoring box in parallel. Relying on the program installed on the host, data interaction between the host and the sensors was carried out based on hexadecimal coding, and the measured values of each sensor were synchronized to the computer.

#### 3.1.3. Test Scheme

In this study, a series of tests was planned to comprehensively investigate the performance under different operating conditions. The test scenarios include upward operating condition, downward operating condition, and bidirectional operating condition, as listed in Table 3. For each of these test scenarios, the speed of the train model was set at about 0.051 m/s, which corresponds to a representative operating speed of 55 km/h within the tunnel. To ensure the reliability and accuracy of the test results, each operating condition will be tested five times. Repeating the tests five times for each scenario can help reduce random errors and obtain more stable and valid data.

### 3.2. Tunnel Model Validation

#### 3.2.1. Fundamental Diffusion Theory

The instantaneous point source solution for pollutant variation within the tunnel can be expressed as [23].

(1) $$c=c_{0}\exp\left[-\frac{{\left(x-ut\right)}^{2}}{4Dt}\right]$$

in which *c* is the pollutant concentration inside the tunnel (mg/m^3^); *c*_0_ is the exhaust concentration of the diesel engine (mg/m^3^); *x* represents the distance from the sampling point to the tunnel entrance (m); *u* represents the wind velocity inside the tunnel (m/s); *t* represents the time (s); and *D* represents the diffusion coefficient of the pollutant (m^2^/s).

#### 3.2.2. Model Validation Results

**Figure 4:**
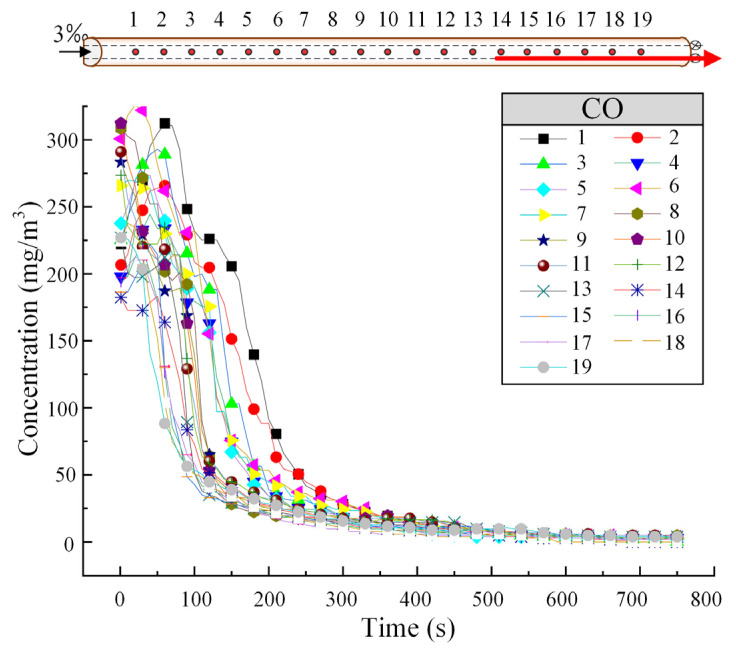
The variation of CO concentration within the tunnel under the downward operating condition of the train. (The red arrow represents the running direction of the train.)

Figure 4 shows the variation in CO concentration at different tunnel cross-sections when the train is descending at a speed of 55 km/h. As shown in Figure 4, CO disperses more quickly at higher concentrations, and the dispersion ability gradually decreases, with the CO concentration at each cross-section tending to stabilize around 300 s.

**Figure 5:**
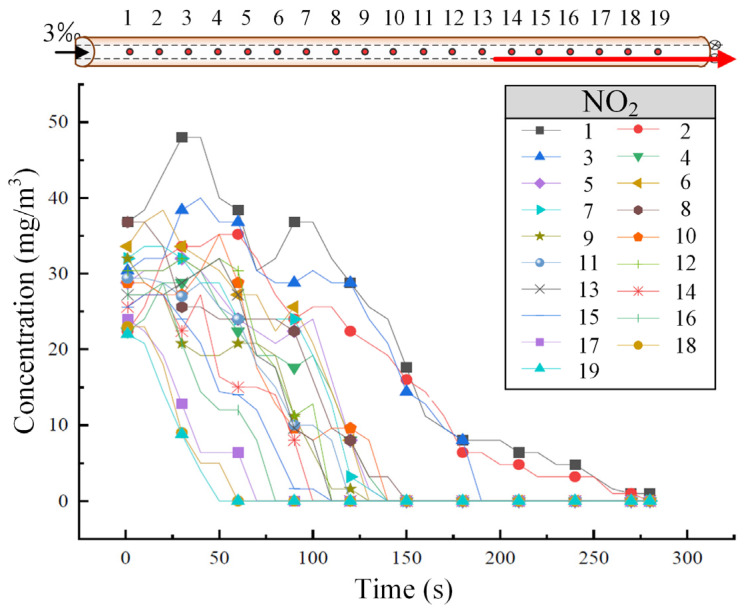
The variation of NO_2_ concentration in the tunnel under the downward operating condition of the train. (The red arrow represents the running direction of the train.)

Figure 5 shows the variation of NO_2_ concentration at different tunnel cross-sections when the train is descending at a speed of 55 km/h. As shown in Figure 5, the concentration decrease pattern of NO_2_ at each sensor is similar to that of CO. NO_2_ primarily disperses toward the high tunnel entrance, but the diffusion rate is slower compared to CO. After approximately 150 s, the concentration of NO_2_ tends to stabilize and become uniform across the cross-sections.

**Figure 6:**
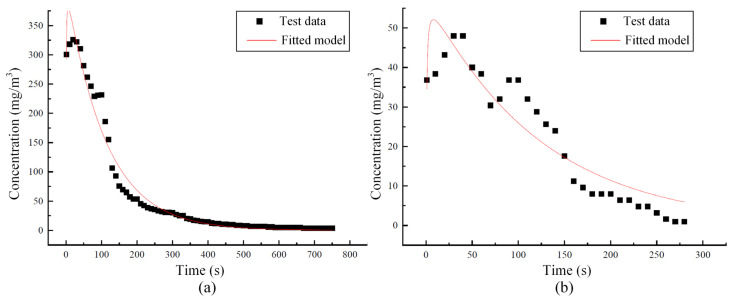
The concentration–time fitting curve of the harmful gas within the tunnel: (**a**) CO; (**b**) NO_2_.

To verify the rationality of the diffusion data above, the relationship between CO and NO_2_ concentrations and time at different positions within the tunnel was fitted based on the instantaneous point source one-dimensional diffusion fundamental solution (Equation (1)), and the R^2^ values during each fitting process were also calculated. The average fitting results of the gas concentration around the NO_2_ sensor are shown in Figure 6. Including the fitting results presented in Figure 6, for CO, all the R^2^ values of the concentration fitting results are greater than 0.91, while for NO_2_, all the R^2^ values are greater than 0.80. These fitting results indicate that the diffusion of CO and NO_2_ concentrations in the scaled tunnel model generally follows the one-dimensional diffusion theory for an instantaneous point source, thereby confirming the validity of the scale tunnel model. Therefore, the scaled model constructed in this chapter can be further used to explore the distribution and diffusion patterns of harmful gas under different operating conditions.

### 3.3. The Diffusion Law of Harmful Gas Within the Tunnel

Similar to Figure 4 and Figure 5, this chapter also presents the monitoring and analysis of CO and NO_2_ concentrations, and the downward operating conditions and the bidirectional traffic conditions of the train were considered.

#### 3.3.1. Upward Operating Conditions

**Figure 7:**
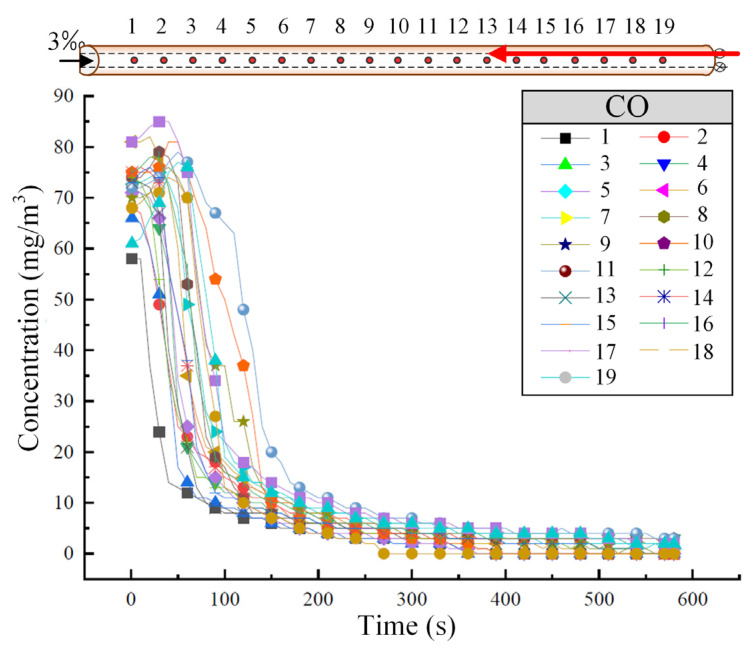
The variation of CO concentration in the tunnel under the upward operating condition of the train. (The red arrow represents the running direction of the train.)

When the train ascends at a speed of 55 km/h through each cross-section of the tunnel, the variation in CO concentration over time and along the tunnel is shown in Figure 7. Similar to the downward operating condition, the CO concentration stabilizes after approximately 250 s. As the harmful gases disperse toward the high tunnel entrance, they also spread toward the low tunnel entrance. During this process, the exhaust gases, influenced by the train’s piston wind, disperse more toward the low tunnel entrance. When the pollutant concentration at each cross-section gradually decreases to about 15 mg/m^3^, the rate of concentration decrease significantly slows down and stabilizes.

A comprehensive comparison of Figure 4 and Figure 7 reveals that the direction of train movement significantly affects the natural diffusion of harmful gas within the tunnel. For a single-track tunnel, when the track slope is aligned with the direction of train movement, it is more favorable for the discharge of harmful gas from the tunnel.

#### 3.3.2. Bidirectional Operating Conditions

**Figure 8:**
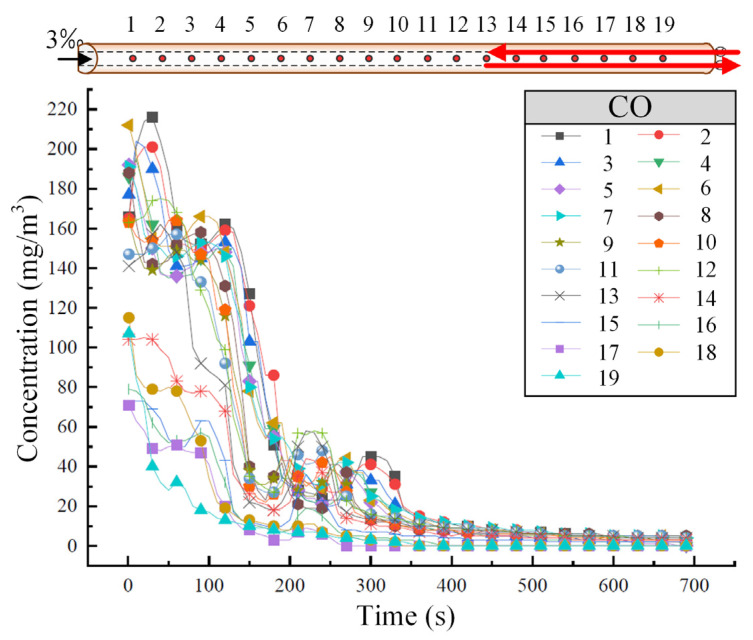
The variation of CO concentration in the tunnel under the bidirectional train operating condition. (The red arrow represents the running direction of the train.)

Figure 8 illustrates the variation in CO concentration at different tunnel cross-sections when two trains travel bidirectionally at a speed of 55 km/h. As can be shown in Figure 8, CO concentration decreases relatively quickly due to the influence of external atmospheric conditions and the piston wind from both the upward and downward driving trains. Near the higher entrance of the tunnel, two sharp drops in CO concentration are observed. This occurs because when external factors facilitate diffusion within the tunnel, CO diffuses more rapidly, whereas when external factors hinder the diffusion of smoke gases, CO accumulates within the tunnel, resulting in localized peaks in concentration. Under the bidirectional operation condition, CO primarily diffuses toward the high tunnel entrance when the concentration is high, although a portion of the CO also disperses toward the low tunnel entrance.

**Figure 9:**
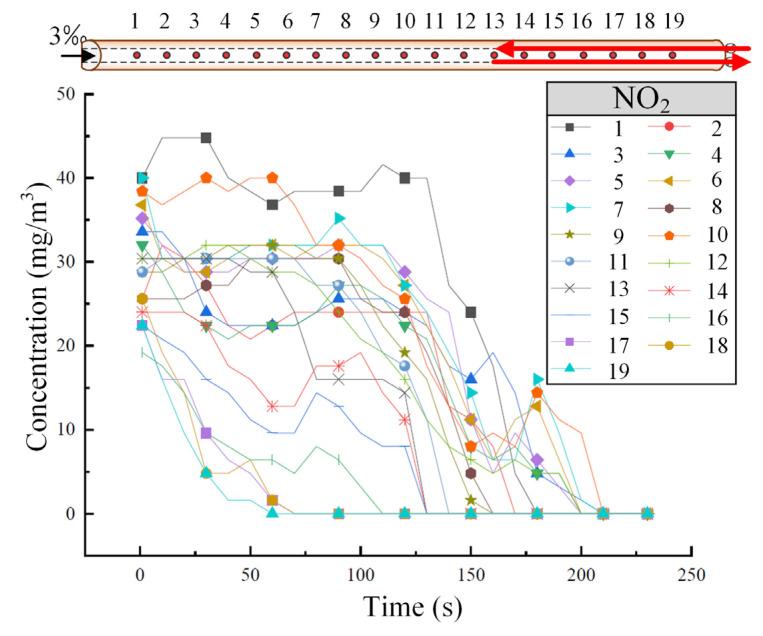
The variation of NO_2_ concentration in the tunnel under the bidirectional train operating condition. (The red arrow represents the running direction of the train.)

Under the same operating conditions, Figure 9 shows the variation in NO_2_ concentration at different tunnel cross-sections. As seen in Figure 9, the decline pattern of NO_2_ concentration is similar to that of CO, but the decrease in NO_2_ concentration is more gradual. However, compared to CO, the fluctuation in NO_2_ concentration along the tunnel is more pronounced under the same operating conditions.

Based on the test results above, it can be concluded that, whether considering bidirectional or unidirectional train operation scenarios, the diffusion of CO and NO_2_ concentrations within the tunnel follows the basic solution of the instantaneous point source one-dimensional diffusion theory. The diffusion of CO and NO_2_ concentrations may either accelerate or be suppressed due to the influence of external factors and the piston wind from both upward and downward moving trains. Under the same operating conditions, CO diffuses faster than NO_2_. Under the downward operating condition, the accumulation of pollutant concentration forms a buildup zone near one-third of the distance from the higher entrance of the tunnel. After bidirectional trains pass each other at different locations within the tunnel, the phenomenon of diffusion toward the high tunnel entrance becomes more pronounced, and a more significant accumulation of harmful gas occurs. In this case, within the specified train interval, the CO and NO_2_ concentrations do not meet the safety ventilation requirements for operation.

## 4. Design of the Tunnel Ventilation

### 4.1. Raw Ventilation Scheme of the Tunnel

**Figure 10:**
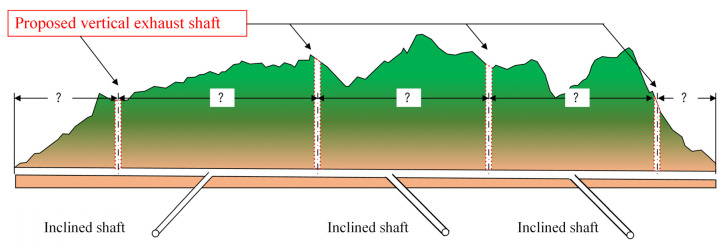
Diagram of the basic tunnel ventilation system.

As previously mentioned, the construction of the tunnel adopted a sectionalized excavation approach for long tunnels using three inclined shafts. Based on the model test results from the previous chapter, it was found that due to the tunnel’s length, harmful gas tends to exhibit a multi-peak distribution along the longitudinal axis of the tunnel. To efficiently achieve rapid ventilation and air exchange within the tunnel, it is necessary to set a vertical shaft between the tunnel entrance and inclined shafts [22], as well as between the two inclined shafts, to assist with ventilation. Inclined shafts are typically established for air inlets, while vertical shafts serve as exhaust outlets, reducing the overall system resistance and maintaining wind pressure balance within the tunnel. Therefore, the basic ventilation scheme for the tunnel is outlined in Figure 10.

### 4.2. Resistance Coefficient Optimization

Due to the influence of the topography and geological conditions at the inclined shaft entrances, the angles at which the inclined shafts intersect with the main tunnel vary, resulting in significant differences in local resistance depending on the direction of airflow. Therefore, it is necessary to optimize the local resistance of the tunnel’s inclined shafts.

According to the requirements of the Code for Design on Operating Ventilation of Railway Tunnel (TB 10068-2024) [39], the local resistance is calculated using the following formula:

(2) $$p_{\xi }=\xi \frac{\rho }{2}\times \frac{1}{F^{2}}\times Q^{2}$$

in which $p_{\xi }$ represents the local resistance (Pa); ξ represents the local resistance coefficient; ρ represents the air density, which is taken as 1.225 kg/m^3^ in this paper; F represents the tunnel cross-sectional area, which is taken as 64.3 m^2^ in this paper; Q represents the tunnel ventilation volume, which is taken as 1.0 m^3^/s in this paper, and it is used for comparing the resistance under the same ventilation volume conditions.

#### 4.2.1. Resistance Optimization for Inclined Shafts

**Figure 11:**
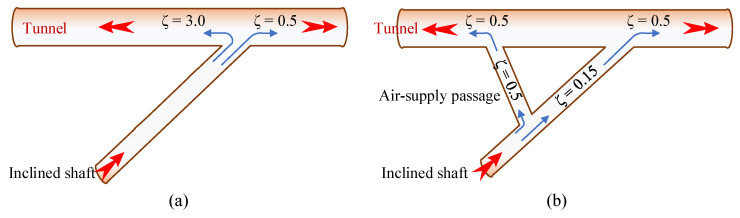
Resistance optimization for inclined shafts: (**a**) original design; (**b**) after optimization. (The red arrows represent the direction of the airflow.)

Limited by the terrain and geological conditions at the entrance of the inclined shaft, the angle of intersection between the inclined shaft and the main tunnel is not 90 degrees. As indicated in “Table A.0.1: Commonly Used Local Resistance Coefficients” in the Code for Design on Operating Ventilation of Railway Tunnel (TB 10068-2024) [39], there is a significant variation in the local resistance for different air supply and exhaust directions in inclined shafts, as shown in Figure 11.

As shown in Figure 11, there is a significant difference in the local resistance coefficients for the same inclined shaft depending on the airflow direction. The local resistance coefficient for large-angle airflow is 0.5, while for small-angle airflow it is 3.0, resulting in a local system resistance of 3.5. Small-angle airflow leads to higher local resistance. To reduce the pressure loss caused by small-angle airflow, this study adds a Y-type air duct to the existing inclined shaft and installs rounded corners (R = d/2, where d is the equivalent diameter of the inclined shaft) at all intersection points to mitigate the additional losses caused by the small-angle intersection between the inclined shaft and the main tunnel.

After the Y-type air duct is added, the local resistance coefficient at the intersection of the inclined shaft and the main tunnel at the inlet end is 0.5, while the local resistance coefficient between the inclined shaft and the Y-type air duct is 0.15, resulting in a total of 0.65. At the outlet end, the local resistance coefficient between the Y-type air duct and the main tunnel is 0.5, and the local resistance coefficient between the inclined shaft and the Y-type air duct is 0.5, giving a total of 1.0.

The local resistance coefficient at the intersection of the inclined shaft and the main tunnel has significantly improved after optimization. Additionally, the local resistance difference for air delivery to both sides of the inclined shaft has been noticeably reduced, which lays a solid foundation for the subsequent development of the sectionalized ventilation scheme.

#### 4.2.2. Resistance Optimization for Vertical Shafts

**Figure 12:**
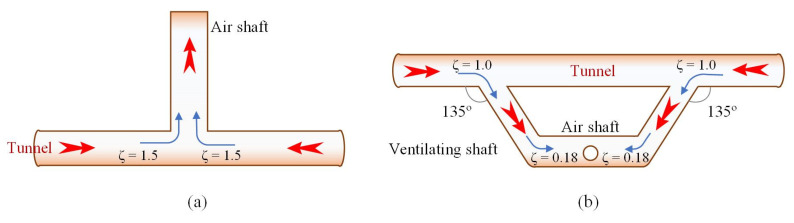
Resistance optimization for vertical shafts: (**a**) traditional design; (**b**) after optimization. (The red arrows represent the direction of the airflow.)

Similar to the inclined shafts, a trapezoidal exhaust duct design was employed for the vertical shafts in this study, as shown in Figure 12. It can reduce the wind pressure loss caused by the traditional vertical shaft ventilation duct’s right-angle exhaust and can also avoid the bidirectional blowing effect on both sides of the vertical shaft ventilation duct. According to “Table A.0.1: Commonly Used Local Resistance Coefficients” in the “Code for Design on Operating Ventilation of Railway Tunnel (TB 10068-2024)” [39], the modification reduces the resistance coefficient of the vertical shaft ventilation duct from 3.0 to 2.36, achieving zonal control of airflow on both sides of the vertical shaft.

#### 4.2.3. Optimized Ventilation Shafts

**Figure 13:**
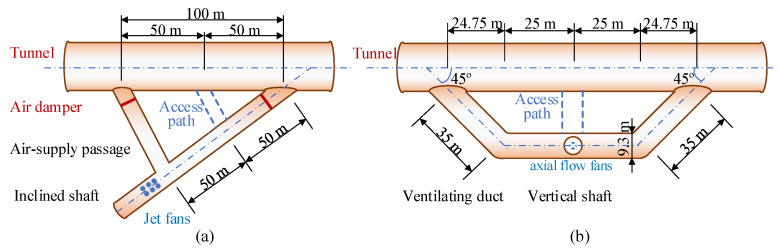
Diagram of the optimization strategy of the (**a**) Y-type inclined shafts and the (**b**) trapezoidal ventilation shafts.

To reduce electrical energy consumption during railway operations, a Y-type ventilation duct is added at the intersection of the construction auxiliary tunnel and the main tunnel, as shown in Figure 13a. It is equipped with a combination of air dampers. The dampers are dynamically adjusted based on the direction of the train and the concentration of harmful gases, altering the direction of the fresh airflow. This design fully utilizes the piston effect of the train to introduce fresh air into the tunnel, thereby reducing the number of ventilation devices that need to be activated and the duration of their operation.

Meanwhile, a trapezoidal ventilation duct is added at the intersection of the vertical shaft ventilation duct and the main structure of the tunnel. The total length of the vertical shaft ventilation duct is 120 m, with an internal slope of +2.5% (60 m) and −2.0% (60 m). The length of the parallel section of the vertical shaft ventilation duct to the main tunnel centerline is 50 m, and the distance between the centerline of the adjacent tunnel and the centerline of the vertical shaft ventilation duct is 24.75 m. The oblique intersection section of the vertical shaft ventilation duct has a centerline length of 35 m and an angle of 45° with the main tunnel.

### 4.3. Validation Results Based on Simulation

In practice, train encounters through tunnel shafts, especially for the trapezoidal ventilation shaft as illustrated in Figure 13b, lead to complex airflow patterns within the tunnel. To further validate the rationale of the optimization of Y-type inclined shafts, a simplified unsteady numerical simulation was conducted in this section.

**Figure 14:**
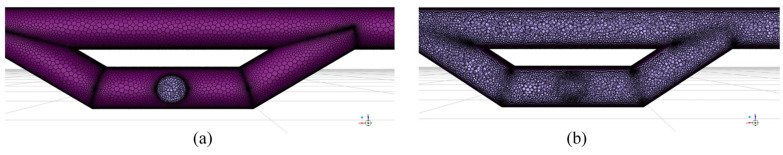
Simulation detail of the ventilation shafts of the tunnel: (**a**) surface mesh generation; (**b**) internal mesh generation.

The pre-processing software Gambit (version 16.0), part of the CFD software suite, is used to divide the computational domain of the tunnel into a grid. The modeling and meshing results are shown in Figure 14. The grid consists of tetrahedral elements, and mesh independence testing is conducted. The model’s boundary conditions are classified into inlet boundary conditions, outlet boundary conditions, and wall boundary conditions. The specific parameter settings are as follows:

(1) Inlet boundary condition: The main tunnel entrance is set as a pressure inlet boundary condition. It is assumed that the pressure at the tunnel exit is equivalent to *P*_2_ = 0, and the pressure at the tunnel entrance is equal to the equivalent pressure difference of the computational model, *P*_1_ = *P*_n_.
(2) Outlet boundary condition: The tunnel exit is set as a pressure outlet boundary condition. Since the pressure at the tunnel exit is assumed to be equivalent to 0, the pressure at the tunnel exit boundary is set to 0. The fluid temperature at the outlet is set to 20 °C and 25 °C, respectively.
(3) Wall boundary condition: The wall is set as a no-slip, adiabatic wall boundary condition. The wall temperature is set to 15 °C, and the wall roughness is set to 0.008 m.

The numerical simulation of the airflow field during the train encounter through the tunnel was conducted using the Fluent module of ANSYS 2024 R1 software. The tunnel flow field was solved using a steady-state implicit method. The turbulence model employed was the RNG k-*ε* two-equation model since it can ensure a certain level of computational accuracy while having relatively low computational costs.

In the CFD simulation, the mesh quality is strictly controlled to ensure the accuracy and reliability of the results. The initial *y-plus* value was set at 30. This value falls within the logarithmic layer range of 30~300, allowing the use of the wall function method to handle wall boundary conditions. It can balance accuracy and efficiency by reducing the number of meshes and computational cost while maintaining a certain level of precision. The polygon mesh division method was selected for mesh generation. This method is suitable for most physical models and performs better in dealing with complex wall models, as it can better fit the geometric shape of the model and reduce mesh distortion. Based on the main structure of the model, the mesh is refined at local positions where airflow diversion occurs to accurately capture airflow changes. Ultimately, the total number of meshes in the model reaches 113,920.

The flow field iteration was performed using the SIMPLE algorithm. To enhance computational accuracy, a second-order upwind scheme was used for discretization. Considering the effect of buoyancy, the airflow region inside the tunnel was defined as FLUD. The energy equation is activated, and then the gravitational acceleration is set to 9.81 m/s^2^.

**Figure 15:**
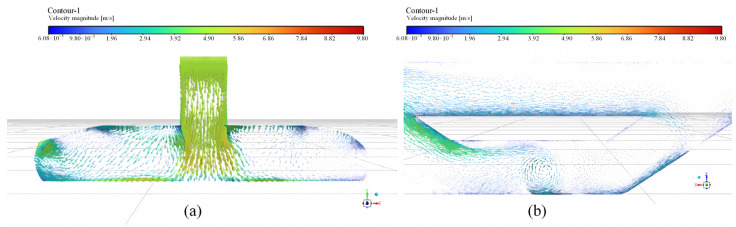
Local velocity vector distribution of the vertical shafts of the tunnel: (**a**) side view; (**b**) top view.

Based on the established simulation model and the adopted computational theory, the simulated velocity distribution of the harmful gas near the vertical shaft and vent duct in the tunnel is shown in Figure 15. It can be observed from Figure 15 that when a train passes through the tunnel near the vertical shaft and vent duct, the flow field inside the tunnel is overall smooth. Although there are minor fluctuations in velocity near the interface transition zones, these do not affect the smooth airflow in the main tunnel structure. The gas velocity increases sequentially from the main tunnel to the vent duct and then to the vertical shaft, which is due to the gradually decreasing design dimensions of the ventilation structures in these areas. The effectiveness of this type of gradient design has already been validated in other studies [40]. This design effectively mitigates the impact of airflow on the main tunnel and train body during operation and facilitates the efficient removal of harmful gases within the tunnel during ventilation. Although the simulation results may not fully match the real airflow conditions, they can preliminarily demonstrate that the combination of a trapezoidal vertical shaft design with a U-shaped vent duct in the connection section between the vertical shaft and the main tunnel is advantageous for optimizing the airflow organization. This optimized design significantly enhances the overall ventilation performance of the tunnel.

### 4.4. Determination of the Ventilation Scheme

#### 4.4.1. Structural Scheme

**Figure 16:**
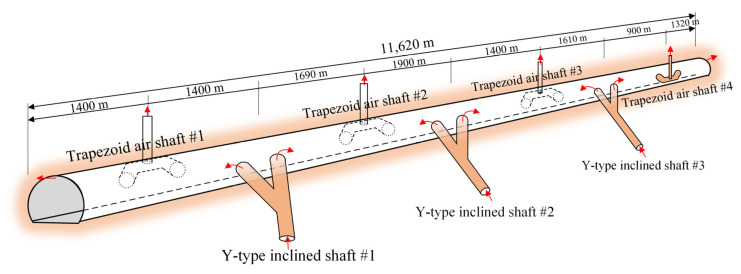
Ventilation system design of the tunnel. (The red arrows represent the direction of the airflow.)

Based on the uniform distribution pattern of harmful gas within the tunnel and the relatively slow attenuation of pollutant concentrations in sections far from the tunnel entrance, combined with the construction of inclined shafts, a sectionalized ventilation scheme for the tunnel studied in this study was determined, as shown in Figure 16. The global ventilation scheme consists of three inclined shafts and four vertical shafts. The goal is to achieve the shortest time for harmful gas to meet the standard within the specified train operating interval. Since the positions of the inclined shafts are fixed, the optimal longitudinal layout distance of the vertical shafts is as follows: vertical shaft #1 is located 1400 m from inclined shaft #1, vertical shaft #2 is located 1600 m from inclined shaft #1, vertical shaft #3 is located 1400 m from inclined shaft #2, and vertical shaft #4 is located 900 m from inclined shaft #3. The detailed parameters are listed in Table 4. Considering factors such as the layout of the vertical shafts, minimum train intervals, airflow velocity within the ventilation ducts and vertical shafts, and the reverse shaft lifting equipment of the site, the ventilation vertical shafts are designed with an effective inner diameter of no less than 3.3 m, with a total of four shafts designed in both main and auxiliary configurations. Additionally, jet fans are set in the inclined shafts to introduce fresh air and oxygen from outside the tunnel, while axial flow fans are used in the vertical shafts to exhaust contaminated air from the tunnel. This ventilation system ensures air quality within the tunnel while enhancing the operation performance of the diesel locomotives.

#### 4.4.2. Energy-Saving Design

(1) Overall design

To further conserve electrical energy, this study proposes the implementation of a smart tunnel ventilation energy-saving control system. The system utilizes methods such as gas detection, train position detection, and tunnel wind velocity and direction monitoring to effectively harness tunnel wind and the kinetic energy of train operation. Through data analysis and automatic learning, the system achieves intelligent operation of tunnel ventilation. It can adjust the corresponding air valves to guide airflow movement based on the wind direction within the tunnel and automatically control the optimal number of fans for ventilation according to train operation frequency and emission levels, maximizing energy-saving operation and maintenance. Additionally, the tunnel intelligent ventilation management control system is equipped with features such as fully automated fan control, management, status monitoring, and safety monitoring, significantly improving the efficiency and safety of tunnel ventilation operation and management. It supports functions like real-time online monitoring and control, fault alarms, maintenance reminders, and data management.

(2) Primary functions

The smart tunnel ventilation energy-saving control system can remotely control the jet fans in the main tunnel, inclined shaft axial fans, and vertical shaft axial fans, as well as coordinate the opening and closing of various air valves. The system is also capable of detecting the position of trains through the train detection system, enabling section-based monitoring of train operations.

The smart tunnel ventilation energy-saving control system can detect the gas concentrations (CO, NO_2_, ozone, dust, temperature, and humidity), wind velocity, and wind direction with the tunnel through the environmental monitoring system. Additionally, the system utilizes sensors and other detection devices installed on the fans to monitor fan vibrations and electrical parameters. This allows for the real-time identification of the operating status of the fan units and the early detection of potential faults, enabling users to stay informed about the equipment’s health status and arrange timely maintenance. It is helpful to minimize losses caused by downtime due to equipment failures.

The smart tunnel ventilation energy-saving control system features data recording, analysis, learning, and intelligent operation. By detecting train positions, gas concentrations, and tunnel wind velocity and direction, the system automatically opens and closes the inclined shaft air valves and vertical shaft air valves. It also automatically optimizes the operation of the main tunnel jet fans and wind direction, as well as the inclined shaft and vertical shaft axial fans, ensuring the most energy-efficient ventilation operation for the tunnel.

(3) Main components

Smart tunnel ventilation energy-saving control system: The smart tunnel ventilation energy-saving control system mainly consists of the tunnel environmental monitoring system, train detection system, integrated safety monitoring and control cabinet for jet fans, integrated monitoring and control cabinet for axial fans, air valve control cabinet, and centralized control system.

Environmental monitoring system: The tunnel environmental monitoring system is composed of a CO sensor, NO_2_ sensor, ozone concentration sensor, dust sensor, wind velocity/direction sensor, and temperature and humidity sensor. Train detection system: The train detection system is composed of a reflective photoelectric sensor, monitoring box, power supply, and communication module. Integrated safety monitoring and control cabinet for jet fans: This consists of sensors, data acquisition and analysis, start-up control module, display module, communication module, and other components. It enables the integrated safety monitoring and operation control of the main tunnel jet fans.

Air valve control cabinet: The air valve control cabinet is responsible for opening and closing the air valves and providing status feedback. Centralized control system: The centralized control system is responsible for the overall tunnel environment monitoring, train operation monitoring, fan and air valve control, fan safety monitoring, and fan fault monitoring, as well as monitoring, management, and intelligent operation and maintenance.

(4) Control strategy

The control strategy is based on the concentration of harmful gases at the exhaust start-up condition, with a minimum start-up time of no less than 30 min. The direction of the main line jet fans, inclined shaft supply fans, and vertical shaft exhaust fans is adjusted according to the tunnel piston wind direction through their respective control cabinets and the opening and closing of air valves. The axial fans operate mainly based on the above-mentioned start-up conditions.

The system employs a fuzzy control strategy to save electricity and extend the lifespan of the fans. It is challenging for the frequent start/stop of fans based on harmful gas concentrations to balance between energy savings and motor protection. The fuzzy control strategy calculates the number of fans to be started in the next cycle based on the previous exhaust record and the time when harmful gas concentrations met the standards. If the time interval is less than 30 min, the number of fans to be started can be optimized. This optimization minimizes energy consumption while maximizing exhaust efficiency. Additionally, the system integrates data from train schedules, time intervals, and quantities to perform comprehensive calculations and records data on various operating conditions of exhaust systems under different train interval times. Through multiple train runs, calculations, and data accumulation, the system can derive the optimal control strategy for the sequential control of fans.

(5) Energy saving calculation

As mentioned previously, an intelligent variable-frequency control strategy was adopted in the ventilation equipment of the tunnel. The parameters of the ventilation equipment inside the tunnel are shown in Table 5. To evaluate the energy-saving potential of the intelligent ventilation system in this tunnel, in this section, two operating conditions are considered, i.e., continuous operation at a constant frequency and operation under intelligent variable-frequency control. The power consumption of the ventilation system under different operating conditions is calculated and analyzed.

1. Continuous operation condition at a constant frequency

Under this condition, consider that all fans operate continuously at the rated frequency (50 Hz) during the train operation period. The railway operation duration is calculated as 16 h per day. Through calculation, the total rated power of the ventilation equipment in the tunnel is 7224 kW, the daily power consumption is 115,584 kWh, and the annual power consumption is 42,188,160 kWh.

2. Operating condition under intelligent variable-frequency control

Under this condition, the rated frequency of all fan motors is 50 Hz. The motor operating frequency is divided into four discrete levels: 10 Hz (lowest frequency), 30 Hz (low frequency), 40 Hz (medium frequency), and 50 Hz (high frequency/rated frequency). According to the principle of the variable-frequency speed regulation of AC motors, the fan shaft power is approximately proportional to the cube of the motor operating frequency. Based on this proportional relationship, the relative power coefficients at each frequency level are calculated as follows: 0.008 (lowest frequency), 0.216 (low frequency), 0.512 (medium frequency), and 1.00 (high frequency/rated frequency).

According to the settings of inclined shafts and vertical shafts in the tunnel (introduced in Section 4.4.1), the tunnel is divided into four relatively independent ventilation control sections. The operating frequency level of the fans in each section is dynamically adjusted according to two key parameters: the number of trains (provided by train detection sensors) and the pollutant concentration (provided by environmental monitoring sensors).

Based on the fact that 160 trains (corresponding to 80 pairs of trains) pass through the tunnel daily, and considering the distribution of trains in the four sections, the system operating states can be discretized into a total of 640 possible operating condition combinations (4 sections × 160 train events). According to statistics, the occurrence frequencies of various types of operating conditions on a typical day are 71 times for the meeting-train operating condition and 569 times for the single-train running operating condition.

(6) Energy-saving effect

Based on the information above, the power consumption under the intelligent variable-frequency control operating condition is calculated by accumulating the energy consumption of all ventilation sections. The energy consumption of each section depends on the number of fans at each frequency level in the section and the duration of the operating condition. According to the calculation results, the daily power consumption under the intelligent variable-frequency control operating condition is 65,437 kWh, and the annual power consumption is 23,884,505 kWh. Compared with the continuous operation condition at a constant frequency, it is reduced by 18,303,655 kWh, and the energy-saving rate is 43.38%.

The above calculation results indicate that under the described typical railway tunnel operating conditions (16 h daily operation with 160 trains passing through), the intelligent variable-frequency ventilation control system based on zoned on-demand adjustment can achieve significant energy consumption reduction compared with the constant operating condition where the fans continuously operate at the rated frequency. The reasons are as follows: (1) Spatial zoning optimization: dividing the tunnel into four independently controlled sections allows the startup or power increase of fans only in the areas where there is a demand, reducing unnecessary equipment operation; (2) cubic law effect of variable-frequency speed regulation: when the fans operate at frequencies lower than the rated frequency (especially in the low- and medium-frequency levels), the power consumption decreases sharply in a cubic relationship as the frequency decreases; (3) dynamic on-demand control: the system precisely adjusts the startup, shutdown, and speed of the fans in each section according to the real-time traffic load (number of trains) and environmental conditions (pollutant concentration), avoiding a large amount of ineffective energy consumption caused by constant full-load operation.

Therefore, adopting an intelligent variable-frequency control system in the tunnel can effectively improve the energy efficiency of the railway tunnel ventilation system and significantly reduce the operating costs.

## 5. Ventilation Effect Validation

### 5.1. Validation Process

**Figure 17:**
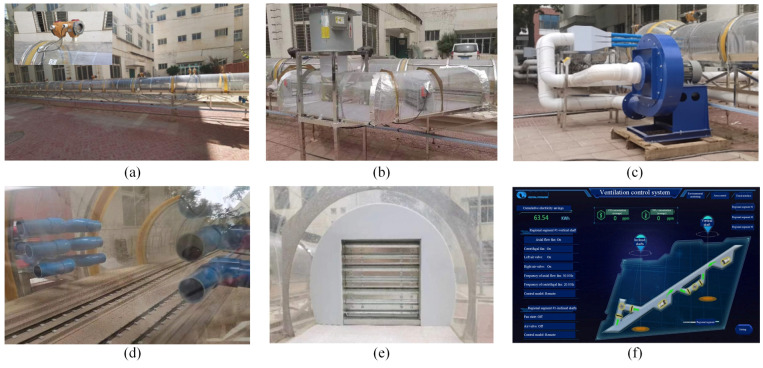
Improved experimental models for ventilation effect verification: (**a**) tunnel model; (**b**) T-type shaft; (**c**) jet fan; (**d**) mechanical ventilation process; (**e**) dampers; (**f**) intelligent monitoring and control system.

To further verify the effectiveness of the proposed ventilation scheme, the tunnel model constructed earlier was improved according to the latest ventilation design, as shown in Figure 17. Lateral inclined shafts and vertical ventilation shafts were added (see Figure 17b), jet fans (see Figure 17c,d) were set up for reducing mechanical ventilation, dampers (see Figure 17e) were set up to meet the needs of different ventilation methods, and finally, the pollutant concentration inside the tunnel was monitored by the intelligent monitoring and system (see Figure 17f), and the jet fans were controlled.

### 5.2. Validation Results

#### 5.2.1. Setting Vertical Shafts and Inclined Shafts Without Mechanical Ventilation

**Figure 18:**
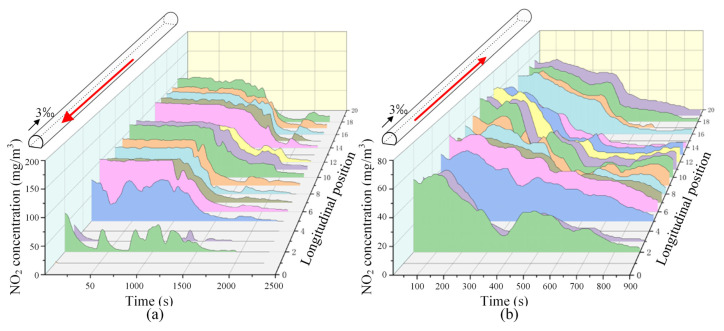
Spatial–temporal distribution of NO_2_ concentration in the tunnel (without mechanical ventilation, under the most unfavorable operating conditions in one direction): (**a**) upward operating conditions; (**b**) downward operating conditions. (The red arrow represents the running direction of the train.)

Figure 18 shows the diffusion process of NO_2_ in the tunnel without mechanical ventilation, and the most unfavorable operating conditions in one direction were considered. As can be seen from Figure 18, after setting vertical shafts and inclined shafts, the NO_2_ concentration decreases uniformly along the tunnel since the harmful gases do not need to be transported to both ends of the tunnel to spread outward. Especially for the harmful gas close to the two ends of the tunnel, its concentration decreases significantly, demonstrating good ventilation performance of the tunnel.

**Figure 19:**
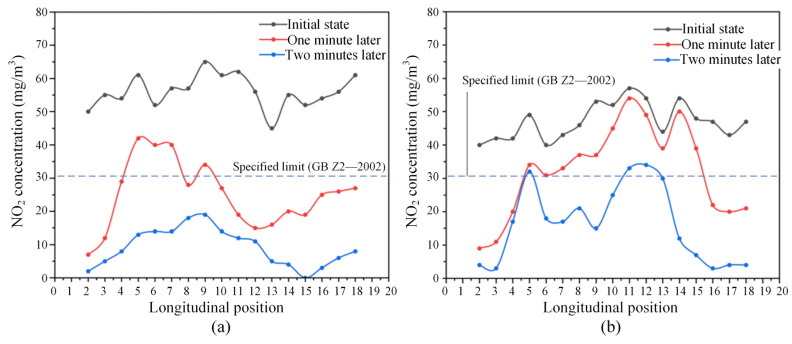
Comparison of the concentration distribution of NO_2_ at different typical moments (without mechanical ventilation, under the most unfavorable operating condition in one direction): (**a**) upward operating conditions; (**b**) downward operating conditions.

Figure 19 shows the comparison results of NO_2_ concentrations at different locations in the tunnel at different typical moments (initial moment, one minute after ventilation, and two minutes after ventilation); the most unfavorable operating conditions in one direction were considered. As can be seen from Figure 19, for both the upward and downward operating conditions, the zone where the NO_2_ concentration decreases more slowly includes the zone of 3.5–4.5 km from the higher entrance of the tunnel, which is located at about 1/3 of the tunnel from the high opening end. This phenomenon is similar to that found in the previous section. It can also be seen from Figure 19 that the downward operating condition is more unfavorable for the dispersion of NO_2_ than the upward operating condition, and the NO_2_ concentration can hardly be reduced to below the safe limit within 2 min, even if all inclined shafts and vertical shafts are opened under this condition.

**Figure 20:**
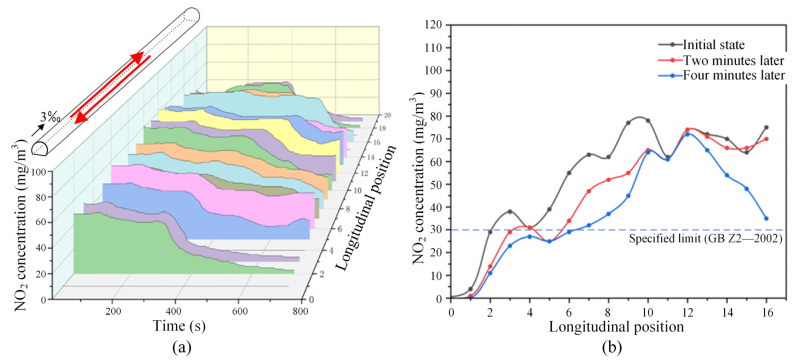
Variation of NO_2_ concentration in the tunnel (without mechanical ventilation, under the most unfavorable operating condition in two directions): (**a**) spatial–temporal distribution of NO_2_ concentration; (**b**) comparison of NO_2_ concentration distributions at different typical moments. (The red arrow represents the running direction of the train.)

Figure 20 presents the variation of NO_2_ concentration distribution within the tunnel under the most unfavorable operating conditions in two directions. Compared to the distribution pattern of NO_2_ observed under one-way operating conditions (see Figure 18), the rate of NO_2_ concentration decay at the midsection of the tunnel is significantly slower. Even after four minutes of ventilation, the NO_2_ concentration in most sections of the tunnel remains above the safety threshold specified in the code. It is indicated that the ventilation efficiency of the vertical shafts and inclined shafts is relatively weak under these operating conditions. Consequently, supplementary mechanical ventilation is necessary to enhance pollutant removal and ensure the air quality within the tunnel.

#### 5.2.2. Setting Vertical Shafts and Inclined Shafts with Mechanical Ventilation

**Figure 21:**
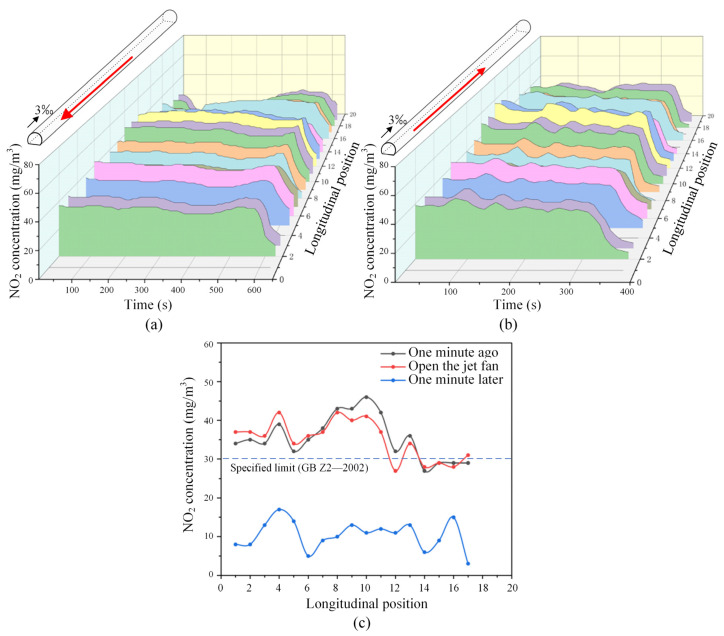
Variation of NO_2_ concentration in the tunnel (with mechanical ventilation, under the most unfavorable operating condition in one direction): (**a**) spatial–temporal distribution of NO_2_ concentration in the upward driving condition; (**b**) spatial–temporal distribution of NO_2_ concentration in the downward driving condition; (**c**) comparison of the distribution of NO_2_ concentration in typical moments of the downward driving condition. (The red arrow represents the running direction of the train.)

Figure 21 depicts the variation of NO_2_ concentrations across different sections of the tunnel under the most unfavorable operating condition for bidirectional traffic. In these scenarios, the mechanical ventilation system was activated. As illustrated in Figure 21a,b, the NO_2_ concentrations in all sections experienced a substantial decrease upon activation of the ventilation fans. Notably, under the downward operating condition, which is depicted in Figure 21c and is more detrimental to ventilation, the NO_2_ concentrations in various tunnel sections reduced to approximately 10 mg/m^3^ after just one minute with the assistance of mechanical ventilation. This value falls below the safety threshold, indicating a significant improvement in ventilation efficacy.

**Figure 22:**
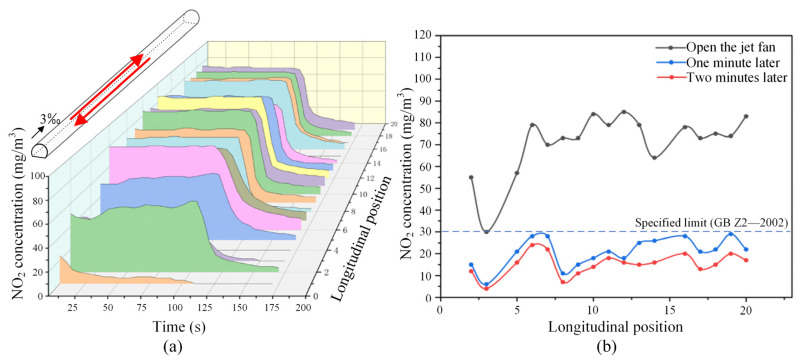
Variation of NO_2_ concentration in tunnels (with mechanical ventilation, under the most unfavorable operating condition in two directions): (**a**) spatial–temporal distribution of NO_2_ concentration; (**b**) comparison of NO_2_ concentration distribution at different typical moments. (The red arrow represents the running direction of the train.)

To further illustrate the effect of mechanical ventilation, Figure 22 presents the change in NO_2_ concentration across various sections of the tunnel following the activation of jet fans, focusing on the most unfavorable operating conditions for bidirectional traffic. As depicted in Figure 22, upon activating the mechanical ventilation system, NO_2_ concentrations in different tunnel sections exhibited a significant decline over a brief period. When compared to the same operating condition without mechanical ventilation (refer to Figure 20b), the NO_2_ concentrations in various tunnel sections can be reduced below the specified safe limits within one minute. Under these circumstances, it is advisable to turn off the fans promptly to conserve energy, as the additional reduction in NO_2_ concentration beyond one minute does not yield substantial benefits. In conclusion, the optimized tunnel ventilation design establishes a solid foundation for efficient ventilation operations, while real-time concentration monitoring serves as a crucial basis for achieving energy-efficient and intelligent tunnel ventilation management.

Vertical ventilation shafts and horizontal ventilation shafts are the two most common types of ventilation shafts in tunnel engineering. Traditionally, these shafts are directly connected to the tunnel, with a relatively simple form. Moreover, the construction of ventilation shafts may affect the control of the alignment accuracy and structural safety during the construction of the main structures of the tunnel.

Based on an actual tunnel engineering case, this paper optimizes the existing construction of inclined shafts to meet the ventilation needs of the tunnel. Meanwhile, a new type of vertical shaft ventilation is also proposed, which significantly reduces the excavation difficulty of both the shaft and the main tunnel while effectively meeting the tunnel’s ventilation requirements. This may provide new ideas for the ventilation design and renovation of similar long tunnels.

## 6. Conclusions

In this paper, the ventilation of an extra-long single-bore double-track railway tunnel is investigated. Firstly, the diffusion law of harmful gas in the tunnel is obtained through scale model tests. Then, based on the resistance coefficient optimization theory combined with numerical simulation, the inclined shafts and vertical shafts of the tunnel are optimized. Finally, the ventilation effect of the tunnel was verified. The main conclusions can be drawn as follows:

1. If the piston wind effect is only considered, the harmful gases in the tunnel primarily diffuse towards higher areas and are then discharged from the high portal end. In contrast, the gases closer to the low portal end tend to diffuse locally towards the low portal for discharge. Under the same operating conditions, NO_2_ diffuses more slowly and is more challenging to discharge compared to CO. When designing ventilation systems for railway tunnels under operating scenarios, NO_2_ can be selected as the key control pollutant for prioritized monitoring and research.
2. Based on the optimization theory of the resistance coefficient, for the railway tunnel analyzed in this study, the resistance coefficient of inclined shafts was reduced from 3.00 to 1.65, while that of vertical shafts was decreased from 3.00 to 2.36. This indicates that the application of the resistance-coefficient optimization theory can effectively optimize the traditional inclined and vertical shafts of a tunnel, leading to a notable enhancement in ventilation efficiency.
3. Based on the analysis in this study, compared with the traditional operating condition where fans run at full frequency throughout the whole period, the intelligent variable-frequency ventilation control system that adjusts on demand based on the tunnel ventilation sections can achieve precise air supply in different sections. It significantly reduces the ineffective operation time of fan equipment and remarkably cuts down the energy consumption of the ventilation system. The annual energy-saving rate can reach 43.38%.
4. Based on the verification results of this study, although the ventilation efficiency of the tunnel has been significantly improved after the optimization of the ventilation ducts, the pollutant concentration inside the tunnel cannot be controlled after four minutes under the most unfavorable operating condition in two directions. After integrating mechanical ventilation, even under the most unfavorable operating conditions, the harmful gases can be reduced below the regulatory safe limits (30 mg/m^3^) within one minute. This indicates that for railway tunnels with high traffic density, it is necessary to combine the optimization of the tunnel ventilation structure with mechanical ventilation equipment to achieve efficient and safe operational ventilation.

In this study, in order to achieve safe ventilation and ensure good ventilation effects for an extra-long tunnel, the ventilation structure of the tunnel was optimized, and the mechanical ventilation equipment was also integrated, considering the most unfavorable operating conditions. However, the probability of the most unfavorable operating conditions in practice is relatively low. In order to fully leverage the ventilation performance of the tunnel structures and save energy consumption as much as possible, further intelligent ventilation design is necessary in future work.

## Figures and Tables

**Table 1:** Emission limit of harmful gas generated from diesel locomotives.

Harmful Gas (g/kW·h)				Smoke and Dust (m^−1^)
CO	NO_x_	PM	HC	
1.5	2.0	0.02	0.46	0.5

**Table 2:** Monthly wind velocity, temperature, and humidity data collection results at the tunnel entrance during the observation period (July 2021–April 2022).

Month	Jul.	Aug.	Sep.	Oct.	Nov.	Dec.	Jan.	Feb.	Mar.	Apr.
Average wind velocity (m/s)	0.66	0.68	0.69	0.72	0.76	0.79	0.78	0.77	0.81	0.84
Average temperature (°C)	28.10	28.20	28.39	28.62	28.91	29.08	29.25	30.40	31.34	31.46
Average humidity (%RH)	66.94	65.18	62.84	58.57	52.49	49.16	49.00	48.65	50.15	31.46

**Table 3:** Introduction of test conditions.

Traffic Scenario	Speed (km/h)	Number of Test Times
Upward operating condition	55	5
Downward operating condition	55	5
Bidirectional operating condition	55	5

**Table 4:** Vertical shaft distribution along the tunnel.

Vertical Shaft Designation	Projection Mileage	Vertical Shaft Depth (m)
Vertical shaft #1	DK149 + 672	205.80
Vertical shaft #2	DK152 + 762	396.18
Vertical shaft #3	DK156 + 062	428.28
Vertical shaft #4	DK158 + 572	431.25

**Table 5:** Parameters of the ventilation equipment of the tunnel.

Fan Types	Rated Power (kW)	Number
Jet fan	45	48
Axial flow fan	132	12
Axial flow fan	185	8
Axial flow fan	220	4
Axial flow fan	280	4
In total	7224 kW	76

## Data Availability

The raw data supporting the conclusions of this article will be made available by the authors on request.
